# Web-Based Psychoeducation Program for Caregivers of First-Episode of Psychosis: An Experience of Chinese Population in Hong Kong

**DOI:** 10.3389/fpsyg.2016.02006

**Published:** 2016-12-26

**Authors:** Sherry K. W. Chan, Samson Tse, Harrison Long Tin Sit, Christy L. M. Hui, Edwin H. M. Lee, Wing C. Chang, Eric Y. H. Chen

**Affiliations:** ^1^Department of Psychiatry, The University of Hong KongHong Kong, China; ^2^State Key Laboratory of Brain and Cognitive Sciences, The University of Hong KongHong Kong, China; ^3^Department of Social Work and Social Administration, Faculty of Social Sciences, The University of Hong KongHong Kong, China

**Keywords:** web-based, psychoeducation, caregivers, psychosis, first-episode

## Abstract

Caregivers of patients with first-episode psychosis are often with little knowledge about the illness and experience more burden of care. Psychoeducation to caregivers has shown to be effective in improving outcomes of patients and possibly reduce stress of the caregivers. This has been recommended as one of the key psychosocial interventions for specific early intervention for psychosis service. However, the accessibility of the service, high case load of the health care professionals and self-stigma of caregivers are often the barriers of implementation of these programs in the real world. Incorporating the convenience of information access via internet and the concept of self-management approach, an interactive internet-based self-help psychoeducation program (iPEP) (www.ipep.hk) for the caregivers of patients with psychosis was established targeting the caregivers of patients with psychosis at early stage in Hong Kong. It provides a comprehensive online resource center in both written and video format on knowledge about psychosis, skills of care, and information of local resources. It also has a forum facilitate information exchange between healthcare professionals and the caregivers, allowing for self-management. Over 800 caregivers have joined the program as members. The evaluation of the subset of the members/users of the iPEP suggested that the iPEP program has been well received and appreciated by the caregivers of patients with first episode psychosis locally. It can potentially serve as a platform for future development of specific manual-based psychoeducation program for caregivers targeting at the needs of different groups.

## Family Intervention of Psychosis: An Overview and Experience in Hong Kong

Psychotic disorders have a prevalence of 3% presenting a major global burden to society (Collins et al., 2011). Informal care by the family members plays a pivotal role in the continued support of people with psychosis including medication adherence, encouraging improvement of social function, and shortening hospitalization duration (Penn et al., 2005; Sin et al., 2005). Based on the critical period hypothesis (Birchwood et al., 1998), the first 2–5 years are critical to the long term outcomes of patients with psychosis. Therefore, the role of caregivers during this period could be even more crucial. Caring for patients with psychosis is demanding and caregivers often experience a high level of distress including depression, anxiety, and subjective experience of burden (Martens and Addington, 2001). One study has found one third of caregivers suffer from depression (Kuipers and Raune, 2000). This is particularly so during the early stage of the illness (Martens and Addington, 2001) when caregivers are often facing shock, grief, and having poor understanding of the illness (Addington and Burnett, 2004). Therefore, family intervention for caregivers of patients with first episode psychosis (FEP) is essential to the long term outcomes of patients and the health of caregivers (Addington et al., 2005).

Since the 1990s, there has been an international effort in development of special early intervention services for first-episode psychosis to improve the long-term outcomes of patients with psychosis (Bertolote and McGorry, 2005) and family focused intervention has been recommended as one of the key psychosocial interventions by the international clinical guidelines (International Early Psychosis Association Writing Group, 2005). There have been various studies providing evidence on the effectiveness of family intervention. It has been reported to be effective in reduce rates of hospitalization, relapses, improved social outcomes, and improved treatment adherence (Mari and Streiner, 1994; Pilling et al., 2002; NICE, 2009; Pharoah et al., 2010). Recent review found that 60% of the studies showed a significant positive impact of the intervention to the caregiver outcomes (Lobban et al., 2013b). Psychoeducation is one of the key elements of family interventions with aims to improve caregivers’ knowledge of the illness, communication skills, and skills of coping of care of their relatives. The effectiveness of family psychoeducation intervention on its own has been demonstrated in reducing relapse, rate of rehospitalisation, and improving functioning (Mari and Streiner, 1994; Barbato and D’Avanzo, 2000; Rummel-Kluge and Kissling, 2008; Jewell et al., 2009; Pharoah et al., 2010; Lucksted et al., 2012). It has also been shown to improve knowledge of caregiver and improve coping of family in a systematic review (Sin and Norman, 2013). However, as psychoeducation is often part of the element of family intervention, it is difficult to distinguish its specific effect. There were only three studies identified in the recent review to explore the effect of psychoeducation on its own (Lobban et al., 2013b). Therefore, recent NICE guidelines stated that the evidence is not sufficient to make recommendation for family psychoeducation for patient care (NICE, 2014). Furthermore, few controlled studies involved caregivers of patients with FEP.

Despite some evidence on the effectiveness of family intervention and family psychoeducation, implementation of these programs in the real world is poor. Partly due to the high case-loads of health care professionals and poor accessibility of the service, partly because of the caregivers’ feelings of stigmatization. Furthermore, competing roles of the caregivers and physical distance could also be barriers to the attendance of the program. A self-management approach in providing psychoeducation to caregivers at the pace and intensity of their choice has been suggested recently (Lobban et al., 2013a) and its feasibility and effectiveness has been studied in caregivers of patients with long term illness (Chiu et al., 2013) and FEP (Lobban et al., 2013a; Chien et al., 2016). This approach has also been recognized as an empowering process to bring about better personal adaptation and improvement of self-efficacy.

By mid-2015, the population of Hong Kong reached 7.3 million (CSD, 2016) and 93.6% are ethnic Chinese (CSD, 2012). Differing from the Western individualistic culture, Chinese culture is a collective one with strong emphasis on harmony, respect for elders and superiors (Bond, 1991). Family is a particularly important source of support and care to patients with FEP in Hong Kong. More than 80% of patients 10 years after their FEP were found to be still living with their family of origin (Chan S.K. et al., 2015). In Hong Kong, an early intervention service, the Early Assessment Service for Young People with Psychosis (EASY) program (Tang et al., 2010), was implemented in 2001 as a territory-wide service within the public healthcare system by the Hospital Authority (HA). This program provides a two-year phase-specific intervention to patients with FEP of age 15 to 25. This was further expanded in 2011 to cover the whole adult age group (15–64) and the service duration was extended to 3 years. Although there has been development of different forms of family intervention for caregivers of patients with FEP as research program (Chien et al., 2016), implementation of the program in the actual settings are limited and was mainly in a format of short-term face-to-face family groups. The similar barriers as mentioned has restricted the participation of the groups. Furthermore, Hong Kong is fast pacing and highly commercialized city. Most caregivers, particularly those of younger patients, often had difficulties in attending the groups because of time and venue restrictions.

In view of the low participations of the psychoeducation groups, telepsychiatry has been suggested as a possible format in delivering psychoeducation to caregivers in Europe. Research has demonstrated its effectiveness in improving knowledge of illness of caregivers (Haley et al., 2011). However, it requires videoconferencing equipment and may have limited its dissemination. Incorporating the convenience of information access via internet and the concept of self-management approach, an interactive internet-based self-help psychoeducation program for the caregivers has the potential to bridge the gaps of service. With this context, Internet-based psychoeducation program for caregivers of psychosis (iPEP)^1^ was established in 2013 to provide self-directed service of seeking information on knowledge about psychosis, caregiving skills and local resources.

## Development of Web-Based Intervention iPEP

In collaboration with several local non-government organizations (NGOs), this internet-based psychosis education program for caregivers (iPEP) was established with an aim to provide up-to-date online information about psychosis and available community resources. The website was designed to cater needs of users of a wide age group based on the experienced generated from the development of websites of psychoeducation for patients (Rotondi et al., 2007). The important sections were highlighted with enlarged font for easy searching.

Information for caregivers of patients with FEP was prepared by the clinicians in collaboration of the multidisciplinary clinical team working in EASY with FEP and was based on the questions raised during the talks for caregivers held previously and the study on the knowledge about psychosis of caregivers (Chan K.W. et al., 2015). This includes 19 articles covering detailed information about psychosis, such as aetiology of psychosis, different treatment modalities, recovery, relapse, medication side effects, and risk management. There are 15 articles providing information specific for skills of caregiving and self-care including communication skills with patients, skills handling common difficult situations (e.g., when the patients refuse to take medication; have poor insight; lack of motivation) and caring for themselves when feeling stressed. **Table 1** shows details of the content. Each of these articles has been recorded into a YouTube video by healthcare professionals including psychiatrists, social workers, and clinical psychologists ranging between 2 and 5 min. Caregivers can pace their own learning schedule with the readily accessible videos in any location. Articles of same content in PDF format is also available for download. This multimedia information resource can facilitate self-learning of caregivers and also be incorporated into the existing psychoeducation group programs. In order to increase the accessibility and cater for people with different language needs, the content of the website, both written and video, were prepared in both Chinese (Cantonese) and English.

**Table 1:** Structure and content of iPEP website.

Basic knowledge	Treatment	Recovery
**Psychosis**		
• What is psychosis?	• What are the treatments for psychosis?	• What is recovery?
• What are the symptoms?	• Can patients with psychosis go to work?	• Can patients with psychosis go to school?
• What is a hallucination?	• How can medication help?	• What are the risk factors for relapse?
• What is a delusion?	• What are negative symptoms?	• How about treatments other than medication?
• What are the stages of psychosis?	• What are the uses and side effects of medication?	
• How is psychosis being diagnosed?	• How can side effects be managed?	
• What causes psychosis?	• Can I stop medications on my own?	

**Tips on caring for patients**		**Caregivers’ self-support**

**Caregiver**	
• Why is family support important?		• What might be caregiver be feeling?
• Suggested skills and attitude toward patients with psychosis?		• What expectations should I have?
• Should I look after the patient long-term?		• Am I stressed?
		• How can I relax myself?
• What should I do if the patient is reluctant to see the doctor or take medications?		• How should I express praise and care?
• What should we do when the patient is experiencing delusions or hallucinations?		• How should I express discontent?
• What should we do when the patient is experiencing delusions or hallucinations?		
• What should I do if the patient lacks motivation?		
• What should we do if the patient is suicidal?		
• What should we do if the patient attempts to harm other people?		
• What are the procedures for involuntary admission?		
**Community resources**	
*Health care and medical services:*		*Occupational Training*
• Early Assessment Service for Young People with Early psychosis (EASY)		• Sheltered Workshop
• Community Psychiatric Nursing services (CPNS)		• Supported Employment
		• Integrated Vocational Rehabilitation Service Centre (IVRS)
• 24-hour Hospital Authority Psychiatric Hotline		• Work Extension Program (WEP)
*Community Support Services*		*Residential Care Services*
• Early Psychosis Foundation (EPISO)		• Supported Hostel
• Integrated Community Centre for Mental Wellness (ICCMW)		• Halfway House
• Other service		• Long Stay Care Home
*Financial Aid*		*Rights*
• Social security allowance scheme (SSA)		• Registration card for Person with Disabilities
• Comprehensive Social Security Assistance (CSSA) Scheme		• Public Transport Fare Concession Scheme

Comprehensive local mental health services provided by both NGOs and government, including services in hospital, vocational trainings, residential care services and financial aids (17 articles), were provided on the websites with contact details (**Table 1**). Any relevant local and international news and activities about psychosis were posted to facilitate the caregivers to seek help and obtain support.

An interactive on-line forum was established to facilitate sharing among the caregivers and communications with the clinicians. Caregivers are encouraged to join as members of the website to form an online community. All members can access the forum of the website where they can post questions that would be answered by the professionals. Responses from other members were welcomed. The website administrator is responsible for the moderating of the forum. In-depth and more specific information about different aspects of psychosis were provided depending on the interests of members. Real stories of caregivers and online interviews of patients were posted on the website. This narrative approach can facilitate learning and sharing with each other.

Since caregivers often value face-to-face group-based sharing (Sin and Norman, 2013), regular psychoeducation talks specifically for caregivers were conducted to provide an off-line interactive platform among the caregivers. Apart from the healthcare professionals as speakers to share knowledge and skills, patients, or caregivers were also invited as speaker to share their first-hand experience.

The iPEP website was promoted among the caregivers to improve their capabilities of using it. It has also been promoted among the health-care professionals to facilitate integration of iPEP with routine care of patients and caregivers. Promotion activities, including poster and on-site promotion, targeted all the EASY clinics and local NGOs particularly centers with services supporting caregivers. Furthermore, newspapers and a major search engine (Yahoo) in Hong Kong were used for publicity.

## Evaluation Process and Outcomes

Over a period of one and half years, 809 caregivers registered iPEP as members. Among the caregiver members, 256 (31.6%) were males and 545 (67.4%) were females with age ranging from 17 to 81 years (*N* = 387, mean = 44.2 years, *SD* = 14.7). Most were recruited at the outpatient clinics of hospitals, some were from the NGOs and some registered online themselves. Using Google analytics^2^, the website had recorded a total of 58333 views from May 2014 to August 2015. On average, 3204 count views were recorded each month with peak in May 2015 reaching 6045 views.

### Member Evaluation of the iPEP

In order to test the usability of the website, 10 caregivers were invited to search for information from the website to answer two questions. All caregivers were recruited from the EASY outpatient clinic. The task was designed to mimic a real-world search based on a previous research (Brunette et al., 2012). These include searching for the side effects of atypical antipsychotics and identifying whether stress is a risk factor of relapse. All of the participants were new to the website without previous orientation. They have all had previous experience in using internet. For each question, the time of finding the right page was recorded. The overall time to search for a specific information on the website was no more than 1 min, suggesting that iPEP is user-friendly and easy for them to locate information they need.

Ten Percentage of the total members (81 caregivers) were randomly invited to participate the interview. Modified questionnaire of the Scales for Perceived Usefulness and Perceived Ease of Use (Davis, 1989) was used to evaluate the usability of the website. The questionnaire consisted of 16 items assessing usefulness of content, ease of use, and usability of some special features (e.g., YouTube learning videos and interactive forum) (**Table 2**).

**Table 2:** Caregiver evaluation on usability of iPEP website over usefulness, ease of use and usability of some general features of the website (*N* = 81).

The iPEP website can..	Strongly agree/Agree	Neutral	Disagree/Strongly disagree
Enhances caregiving skills	55 (69.6%)	18 (22.8%)	6 (7.6%)


Enhances knowledge on psychosis	69 (85.2%)	9 (11.1%)	3 (3.7%)


Increases understanding of local resources	62 (77.5%)	12 (15%)	6 (7.5%)


Enhances confidence to take care of patients	47 (58.8%)	28 (35%)	5 (6.3%)


Makes me feel supported	59 (74.7%)	17 (21.5%)	3 (3.8%)


I would recommend the website to others	66 (83.5%)	11 (13.9%)	2 (2.5%)


Is easy to use	61 (75.3%)	12 (14.8%)	8 (9.9%)


Is easy to learn how to operate	64 (80%)	11 (13.8%)	5 (6.3%)


Is clear and easy to understand	66 (81.5%)	11 (13.6%)	4 (4.9%)


Has abundant contents	60 (74.1%)	14 (17.3%)	7 (8.6%)


Has a deep content	55 (67.9%)	19 (23.5%)	7 (8.6%)



	**Very useful/Useful**	**Neutral**	**Very useless/Useless**

Information on knowledge of psychosis	63 (77.8%)	16 (19.8%)	2 (2.5%)


Information on caregiving skills	39 (49.4%)	37 (46.8%)	3 (3.8%)


YouTube learning videos recorded by healthcare professionals	47 (66.2%)	23 (32.4%)	1 (1.4%)


Online forum	42 (57.5%)	25 (34.2%)	6 (8.2%)


Updates on activities	52 (66.7%)	22 (28.2%)	4 (5.1%)

Participants reported that they had browsed the website 2–5 times on average. The duration was around 5–30 min. 22.5% were males and 77.5% were females with age ranged from 23 to 63 years (mean = 45.4 years). A majority of the interviewed caregivers reported that the website enhanced their knowledge about psychosis (85.2%); increased their understanding of local resources (77.5%) and made them feel supported (74.7%). Over 80% of the respondents would recommend the website to others. There was a general agreement that the website is easy to learn how to operate (80%); has clear and easily understandable contents (81.5%) and contains sufficient information (74.1%). The participants also rated the usefulness of the general features and they found that information on knowledge of psychosis (77.8%), updates on activities related to psychosis (66.7%) and YouTube learning videos recorded by healthcare professionals (66.2%) are particularly useful. Some participants suggested that the website could have more sharing from other caregivers and they enjoyed interaction with other caregivers during the off-line educational talks organized as part of the iPEP program.

## Future Directions

The effectiveness of psychoeducation of caregivers, with elements of knowledge provision and skills sharing, have been demonstrated extensively. However, the implementation of the program in the real world setting has encountered various barriers because of the different needs of caregivers. A self-management approach in providing psychoeducation to caregivers allows for flexibility and active learning. With the increase in the popularity of internet use, the development of web-based iPEP program provides a comprehensive online resource center with communication forum to facilitate information exchange between healthcare professionals and the caregivers, allowing for self-management. Though the iPEP website has been demonstrated to be user-friendly, the small sample recruited for the test of the usability of the website might have limited the generalizability of the result. The evaluation of the subset of the members/users of the iPEP suggested that the iPEP program has been well received and appreciated by the caregivers of patients with FEP locally.

However, the program has some limitations. The content of the information provided in the website were based on the questions raised during the talks for caregivers held previously and the study on the knowledge about psychosis of caregivers (Chan K.W. et al., 2015). Views of caregivers were not obtained systematically for the website development. This could be further improved by obtaining feedback on the content from the caregivers on a regular basis so that the website could be improved continuously. Furthermore, there has not been a comprehensive feasibility test conducted before the launch of the program. The acceptability of using internet, particularly internet forum may vary with different age groups, and different social settings. Some might still not be very familiar with computer and internet and do not have habit of computer using, and hence the main support comes from a more traditional form of interventions such as educational talks and other face-to-face activities. Although, with the exponential increase of the popularity of internet and smart phone, internet will increasingly be an important platform for the newer generation caregivers to seek support and obtain information, level of flexibility should be introduced in the development of the service. Therefore, a hybrid model with both on-line and off-line psychoeducation services may be able to cater for the diversify needs of the caregivers. The current iPEP program can be considered as a pilot project to introduce internet-based program for caregiver intervention. It may serve as a platform for future development of specific manual-based psychoeducation programs with both on-line and off-line sessions for caregivers or various specific self-help on-line programs targeting at the needs of different groups. Further comprehensive evaluation should be conducted to understand the acceptability of such intervention format to the caregivers and to further improve the current platform. In fact, web-based interventions have been demonstrated as an effective form of intervention in many psychiatric conditions. Studies has shown that it is effective in smoke cessation (Strecher et al., 2005; Victor et al., 2015), peer support among patients with schizophrenia (Kaplan et al., 2011) and depression (Griffiths et al., 2009).

Needs of caregivers are diverse and a single form of intervention would be unlikely to be sufficient to all caregivers. Most of the caregivers receive services from different organizations and some with specific caseworkers. Therefore, integration of the program with the existing service is an essential part of the program implementation. Close collaboration with the local service providers and NGOs would be important. Future research should be focused on the assessment of the acceptability of the web-based psychoeducation program, developing strategy of integration of the existing service, and controlled studies should be conducted to evaluate on the effectiveness of on-line based psychoeducation program.

## Ethics Statement

The current study was approved by the HKU/HA HKW institutional Review Board. Written or verbal consent have be obtained from the participants.

## Author Contributions

All authors contributed in the development and establishment of the project. SC contributed to the content of the project, planning, and conducting of the evaluation, preparation of the manuscript. ST contributed to the preparation of the manuscript. HS contributed to the analysis of the data. All authors contributed in the interpretation of the results. All authors contributed revision and editing of the manuscript.

## Conflict of Interest Statement

The authors declare that the research was conducted in the absence of any commercial or financial relationships that could be construed as a potential conflict of interest.

## Footnotes

1 www.ipep.hk

2 http://www.google.com/analytics/
